# Sympatry Increases Predation Risk for an Endangered Ungulate

**DOI:** 10.1002/ece3.74160

**Published:** 2026-08-07

**Authors:** Daniel Gordon, Rebecca L. Lewison, Randy Botta, Winston Vickers, Fernando Najera, Megan Jennings

**Affiliations:** ^1^ Department of Biology San Diego State University San Diego California USA; ^2^ California Department of Fish and Wildlife San Diego California USA; ^3^ Wildlife Health Center University of California Davis California USA

**Keywords:** endangered species, *Odocoileus hemionus*, *Ovis canadensis nelsoni*, predation risk, *Puma concolor*, sympatry

## Abstract

Predation risk for depleted prey populations is known to be influenced by prey abundance. However, predation risk may also be influenced by the abundance of sympatric prey species, particularly when the sympatric species is abundant. This suggests that predation risk estimates for low‐abundance prey may also need to account for the abundance of sympatric species. To understand how sympatry between two prey species with differing abundances affects predation risk, we used an unstudied system in arid Southern California where the co‐occurrence of federally Endangered Peninsular bighorn sheep ( *Ovis canadensis nelsoni* ) and southern mule deer ( *Odocoileus hemionus fuliginatus* ) is thought to affect the predation risk bighorn experience from pumas ( *Puma concolor* ). We characterized predation risk for bighorn and deer across their ranges to understand how topography, habitat, forage, and prey habitat suitability affect where pumas are likely to kill both species. The presence of the second ungulate species increased predation risk for both species, but had the greatest effect on bighorn, with predation risk strongly increasing with deer habitat suitability. The importance of deer habitat suitability to bighorn predation risk confirmed how spatial overlap between low and high abundance prey species can lead to increased predation risk, particularly for lower density ungulate populations. Our results provide more accurate estimates of bighorn predation risk, which is critical to the success of ongoing population recovery, and demonstrate how the density of sympatric species can be considered in population models and management.

## Graphical abstract

We characterized predation risk for Peninsular bighorn sheep and southern mule deer across their ranges to understand how topography, habitat, forage, and prey habitat suitability affect where pumas are likely to predate both species. The presence of the second ungulate species increased predation risk for both species but had the greatest effect on bighorn, with predation risk strongly increasing with deer habitat suitability. Our results provide more accurate estimates of bighorn predation risk, which is critical to the success of ongoing population recovery and demonstrate how the density of sympatric species can be considered in population models and management.

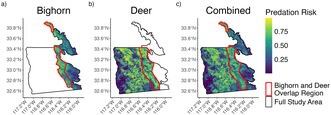

## 1 Introduction

Predation is a primary cause of mortality for ungulates (Cristescu et al. 2022; Kiffner and Lee 2019) and is known to have a significant impact on ungulate populations through both direct mortality (Cristescu et al. 2022; Kautz et al. 2022; Kiffner and Lee 2019; Pöllänen et al. 2023) and indirect, non‐consumptive effects (Chitwood et al. 2022). However, predation risk is not uniform across the landscape. Risk may reflect biotic factors, such as prey density, as well as abiotic habitat features (e.g., habitat type and topography), which impact a predator's ability to hunt and the ability of prey to detect or escape a predator (Gaynor et al. 2019). This spatial variance creates a dynamic predation risk mosaic with patches of high and low risk distributed across the landscape (Gaynor et al. 2019).

Spatial heterogeneity in predation risk can be further influenced by the density of multiple, sympatric prey species, which can alter risk through apparent competition (Holt and Bonsall 2017). Apparent competition is an indirect interaction in which two prey species negatively impact one another through a shared predator (Holt 1977) and has been identified in multiple predator‐ungulate systems across a range of ecosystems (e.g., woodland caribou ( *Rangifer tarandus caribou* ), moose ( *Alces alces* ), and wolves ( *Canis lupus* ), Sierra bighorn sheep ( *Ovis canadensis sierrae* ), mule deer ( *Odocoileus hemionus* ), and mountain lions ( *Puma concolor* ), zebra ( *Equus burchelli* ), hartebeest (*Alcephalus busephalus*), and lions ( *Panthera leo* ), and Przewalski horses ( *Equus ferus przewalskii* ), red deer ( *Cervus elaphus* ), and wolves; Wittmer et al. 2005; Wittmer et al. 2007; Serrouya et al. 2015; Johnson et al. 2013; Ng'weno, Ford, et al. 2019; Ng'weno, Buskirk, et al. 2019; Van Duyne et al. 2009). Negative effects from apparent competition can manifest through either numerical or behavioral responses from predators (Holt and Bonsall 2017). Numerical responses occur when predator abundance increases in response to a greater availability of one prey species, thereby elevating predation pressure on the second prey species (Frenette et al. 2020; Holt 1977; Holt and Bonsall 2017; Serrouya et al. 2015; Wittmer et al. 2005; Wittmer et al. 2007). Behavioral responses can occur when predators preferentially select areas with two sympatric prey species due to greater overall abundance and availability, thereby increasing risk of population decline for one or both species (Dellinger et al. 2020; Holt and Bonsall 2017). Apparent competition can also result in neighborhood effects, when the presence of multiple prey species within a predator's home range impacts the species‐specific risk of predation (Ng'weno, Ford, et al. 2019). For example, when predators preferentially hunt in areas with higher primary prey density and opportunistically feed on alternative prey, a secondary prey species in proximity to the primary prey species can experience higher than expected predation risk relative to their abundance (Cristescu et al. 2019; Ng'weno, Ford, et al. 2019).

Apparent competition is particularly influential when one of the two prey species is significantly less abundant than the other (Frenette et al. 2020; Johnson et al. 2013; Serrouya et al. 2015; Wittmer et al. 2005, 2007). For depleted prey populations, the increased predation risk from apparent competition has been found to have deleterious effects (DeCesare et al. 2010; Johnson et al. 2013; McLellan et al. 2010), resulting in inverse density‐dependent mortality for the less abundant species and, in some systems, local extirpation (DeCesare et al. 2010; Sinclair et al. 1998; Wittmer et al. 2005). Thus, it is important to understand how the presence of sympatric prey species with differing abundances affects predation risk, particularly for the less abundant species (Ng'weno, Ford, et al. 2019).

In the Peninsular Ranges of Southern California, a single apex predator, the mountain lion ( *Puma concolor* , hereafter puma), often preys on two large ungulates, southern mule deer ( *Odocoileus hemionus fuliginatus* ; hereafter deer) and Peninsular bighorn sheep ( *Ovis canadensis nelsoni* ; hereafter bighorn). Pumas preferentially prey on deer (Clapp et al. 2022; Robins et al. 2019), but also on bighorn (Karandikar et al. 2022), with over 50% of collared bighorn mortalities since 1993 attributed to puma predation (Colby and Schaeffer 2023). Deer are relatively abundant throughout their range, with densities around 2 individuals/km^2^, while bighorn are a federally and state endangered population with an abundance around 750 total individuals, creating a clear discrepancy in the population sizes of both species (Murphy et al., *In Review*; CDFW, *personal communication*). This prey–predator dyad provides an ideal system to study how sympatry with a more common species (deer; Botta 2017; Curtis et al. 2023) can affect predation risk for a low‐density species of conservation concern (bighorn; U.S. Fish and Wildlife Service 2000; Colby and Schaeffer 2023).

The impact of apparent competition and neighborhood effects on predation risk remains poorly studied (but see Ng'weno, Ford, et al. 2019), despite the many predator–prey systems that contain multiple overlapping prey species with variable abundance (Atwood et al. 2009; Gervasi et al. 2013; Owen‐Smith 2019). While multiple studies have focused on the effects of apparent competition on mortality rates and abundance, very few have addressed its effects on spatially heterogeneous predation risk (but see Ng'weno, Ford, et al. 2019). Here, we assess the effect of sympatry between bighorn and deer on estimates of predation risk for both species. Although puma predation is listed as a significant threat in the bighorn recovery plan (Colby and Schaeffer 2023; U.S. Fish and Wildlife Service 2000), there has been no quantitative assessment of the impact of deer abundance on bighorn predation risk.

In this study, we assess how puma predation risk for deer and bighorn varies relative to habitat features and population density of both prey species. We then compare predation risk in areas of species co‐occurrence to areas where each species is present individually to determine the impact of neighborhood effects on predation risk. Understanding the interaction between predation and the presence of more common sympatric species is needed to effectively manage and facilitate population recovery for low‐density prey such as the bighorn sheep.

## 2 Methods

### 2.1 Study Area

**FIGURE 1:**
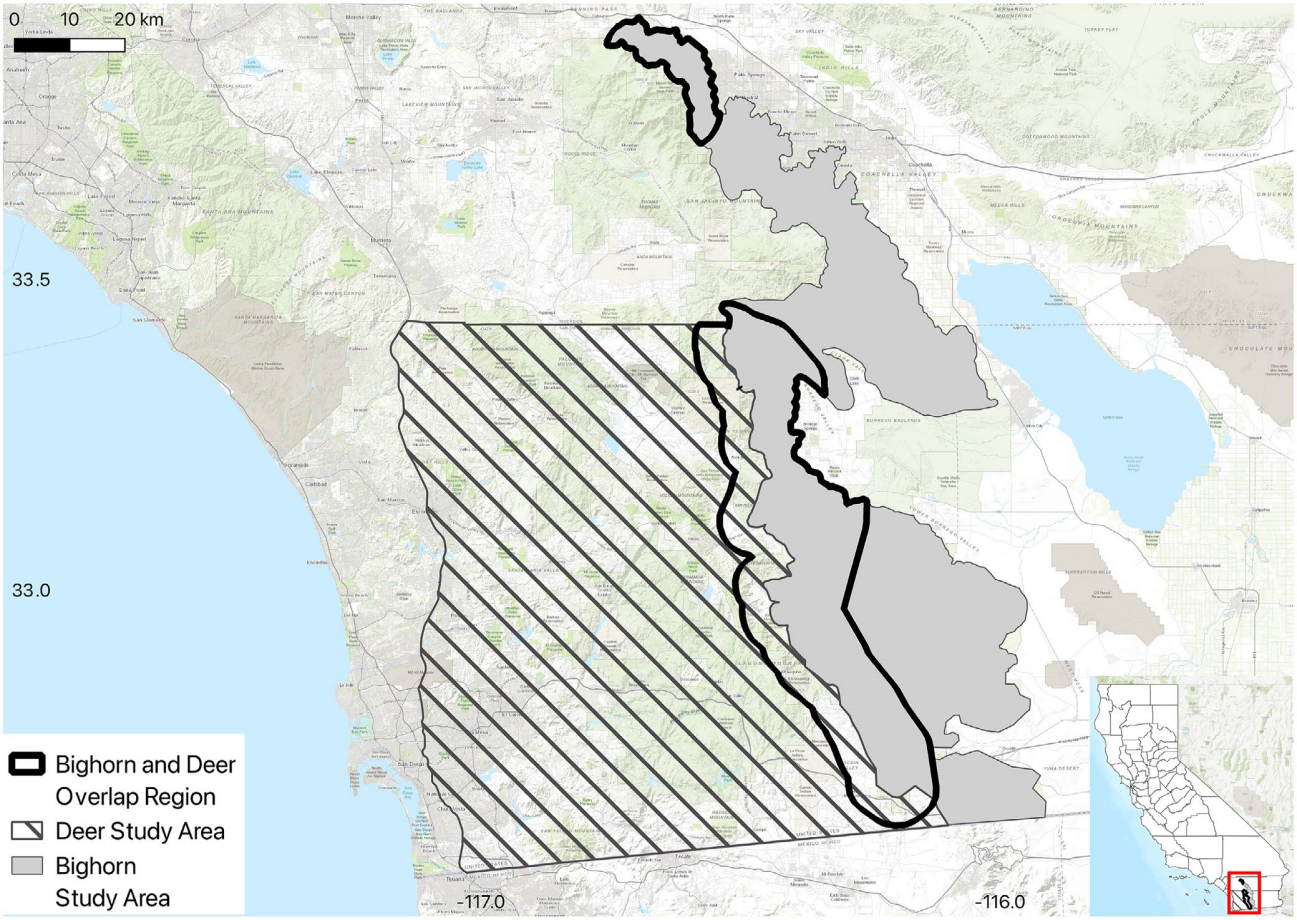
The eastern (bighorn study area) and western (deer study area) sections of our study area and the area of bighorn and deer overlap.

Our study area extends across three counties of Southern California, USA (Riverside, San Diego, and Imperial County). We divided these counties into eastern and western sections based on the distributions of the two prey species. In the eastern section, nine U.S. Fish and Wildlife Service‐designated bighorn recovery regions run southward along the eastern edge of the Peninsular Ranges from near Palm Springs and Mount San Jacinto in Riverside County to the U.S.‐Mexico border in Imperial and San Diego County, covering 3400 km^2^ (U.S. Fish and Wildlife Service 2000). The western section encompasses land west of the bighorn recovery regions and east of Interstate‐15 (Figure 1), where bighorn sheep are absent, but deer occur, an area of 6400 km^2^.

The eastern section of the bighorn recovery regions has elevations ranging from 0 to 2000 m with moderate to steep slopes and mostly rugged terrain. The majority of the vegetation is desert scrub with some areas of conifer woodland at higher elevations (> 1400 m) and small patches of shrubland and barren rock. This area is very hot with large seasonal variation in temperature, with average summer temperatures around 33°C and average winter temperatures around 15°C (National Weather Service 2025). This region is very dry, with annual precipitation ranging from < 20 to 300 mm, with an average rainfall of around 110 mm (National Weather Service 2025). Most of the precipitation falls during the wet season (November–April), with almost none during the dry season (May–October). Artificial water systems called guzzlers or drinkers provide supplemental year‐round access to water in certain recovery regions.

Elevation within the western section of the study area ranges from 5 to 2000 m. The predominant vegetation is shrubland with forest and woodland in the higher elevation mountains. This region has a Mediterranean climate with average summer temperatures around 24°C and average winter temperatures around 12°C (National Weather Service 2025). Precipitation varies in a similar fashion to the eastern study section, with a wet season occurring from November–April and a dry season from May–October. However, total precipitation in the western region is substantially higher, with annual precipitation ranging from 11 to 70 cm with an average of 35 cm at lower elevations, and 33–150 cm with an average of 66 cm in the higher elevation areas (National Weather Service 2025).

### 2.2 Study Species

Southern mule deer are one of six subspecies of mule deer ( *Odocoileus hemionus* ) found in California. Deer are endemic to the region with a geographic range that extends from south of Los Angeles County down into Baja California, Mexico, and from the Pacific coast to the eastern slopes of the Peninsular Ranges and western portions of Anza Borrego State Park in east San Diego County. Managed as a game species by the California Department of Fish and Wildlife (CDFW), they are listed as a species of conservation concern in the San Diego Multiple Species Conservation Program due to habitat fragmentation and limited genetic diversity. However, deer are relatively abundant within their range (Botta 2017; Curtis et al. 2023). Deer are often found in various scrub and chaparral types (e.g., chamise chaparral, semi‐desert chaparral, Diegan coastal sage scrub, montane buckwheat scrub, and acacia scrub) and select areas with greater forage availability (as measured by the Normalized Difference Vegetation Index, NDVI), steeper slopes, and lower elevation (Tomaszewski et al. 2022).

Peninsular bighorn sheep are a federally endangered distinct population segment of desert bighorn sheep (*O.c. nelsoni*) under the federal Endangered Species Act and are listed as a threatened population under the California Endangered Species Act (U.S. Fish and Wildlife Service 2000). Bighorn face numerous threats, including habitat loss and fragmentation, human development, disease, and predation (U.S. Fish and Wildlife Service 2000). Bighorn inhabit the Peninsular Ranges from Mount San Jacinto in Riverside County south into Baja Mexico. Found on the desert‐facing eastern slopes below elevations of 1400 m (Jorgensen and Turner 1975; U.S. Fish and Wildlife Service 2000), bighorn tend to avoid areas of dense vegetation, such as chaparral, and rely on steep, rugged mountainous terrain as escape terrain (U.S. Fish and Wildlife Service 2000).

Pumas are found across California, mainly in forests, shrublands, and mountain habitats (Dellinger et al. 2020). Their primary prey is mule deer; however, as generalists, they also feed on other large mammals, a variety of small mammals, and occasionally birds (Allen et al. 2015). In Southern California, pumas are listed as a threatened population under the California Endangered Species Act due to habitat loss, habitat fragmentation, and the isolation of small populations due to expanding human infrastructure.

### 2.3 Modeling Framework

**FIGURE 2:**
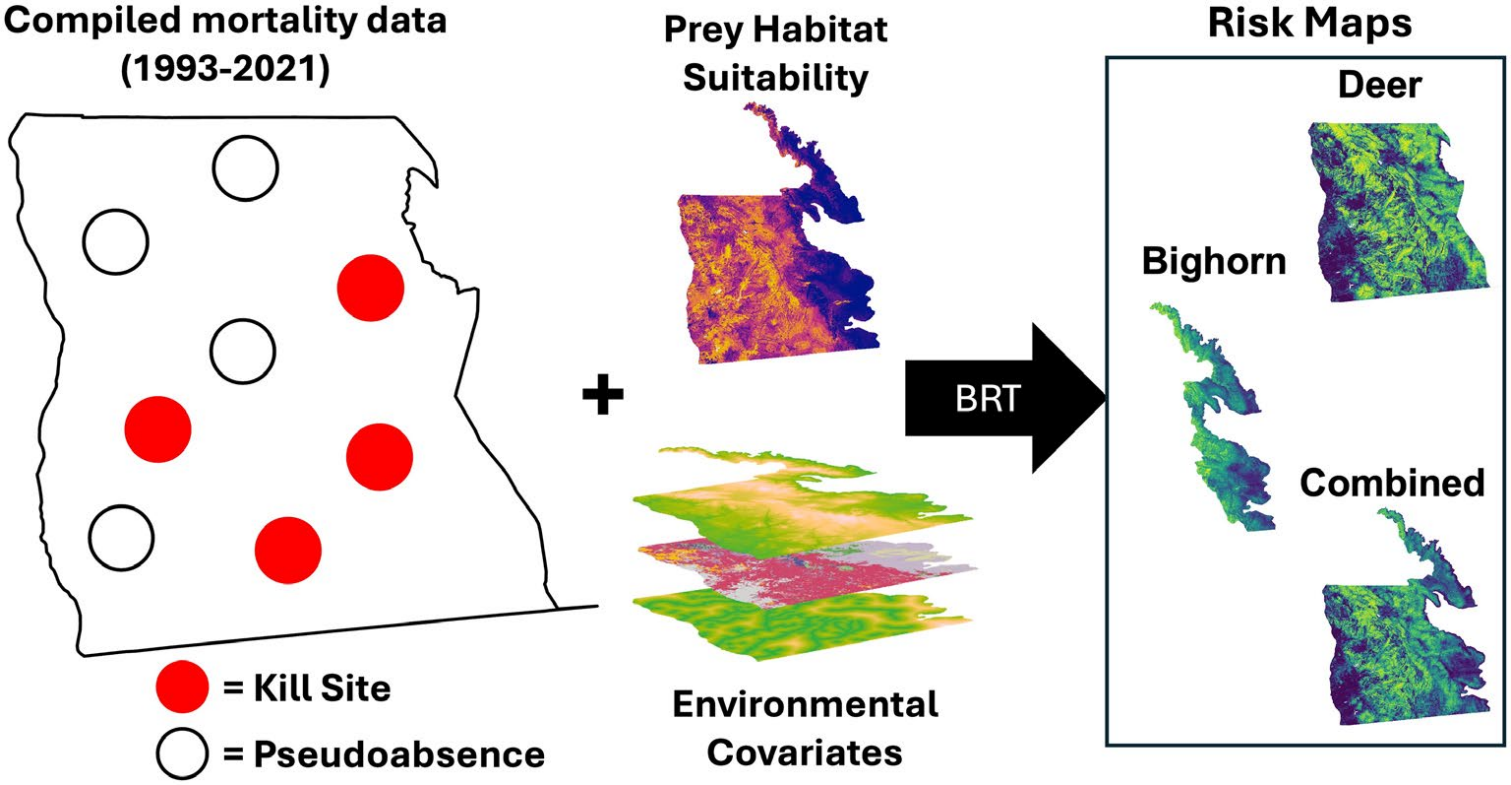
Methodological overview for predation risk models. Mortality data were compiled from 1993 to 2021 and paired with pseudoabsences. Mortality data and pseudoabsences were combined with prey habitat suitability, which was determined using independent field observation data and environmental covariates, and then run through boosted regression trees to create predation risk maps across the landscape.

We used boosted regression trees (BRTs), a machine learning model that can integrate complex data and mixed data types using decision trees and iterative model building to increase model accuracy (Elith et al. 2008), to compare predation risk across the study area using the *flexsdm* package (R Core Development Team 2024; Velazco et al. 2022). We created three BRTs to estimate predation risk maps, using kill site locations, environmental covariates, and prey habitat suitability as predictor variables (Figure 2). The first model estimated bighorn predation risk across the bighorn recovery regions. The second model estimated deer predation risk across the deer range. The third model estimated the combined predation risk for both bighorn and deer across the entire study area.

### 2.4 Kill Site Data

We calculated predation risk for deer and bighorn using three independent kill site datasets to ensure we had a sufficient sample size that covered the entire study area. Similar to numerous previous studies (Prugh et al. 2019; Cristescu et al. 2019; Ng'weno, Ford, et al. 2019; Blake and Gese 2016), we assumed the spatial pattern and presence of kills reflect predation risk, serving as a proxy for predation risk. Dataset 1 consisted of known puma kills confirmed through CDFW field investigations of collared deer and bighorn mortalities from 1993 to 2021. Cause‐specific mortality was classified through standardized protocols as “confirmed”, “probable”, or “possible” puma predation based on field signs, such as bite marks, throat hemorrhaging, caching, and puma scat or tracks at the kill site (Colby 2012a, 2012b). Dataset 1 contained 173 bighorn and 60 deer kill sites.

Dataset 2 was from field investigations conducted by the University of California, Davis (UC Davis) and CDFW of kills by 17 GPS‐collared pumas from 2002 to 2011 using the same standardized protocols as Dataset 1. These sites were designated as kills when uncollared deer or bighorn were found during investigations of puma GPS clusters. Dataset 2 contained 38 bighorn and 82 deer kill sites.

Dataset 3 consisted of putative kill sites predicted through spatial cluster analysis from 65 GPS‐collared pumas monitored by UC Davis and CDFW between 2001 and 2020 (sensu Clapp et al. 2022). We subset the 65 pumas down to 23 pumas that had GPS success rates ≥ 50% and fix rates of four hours or less, while minimizing home range overlap, to ensure kill sites could be reliably identified and would be distributed across the study area. We removed puma locations with a dilution of precision value of > 5 to improve GPS cluster accuracy and reduce error (Lewis et al. 2007). GPS clusters were defined as groups of points within ≤ 100 m of each other, with each point occurring within 1 day of the previous point, as this spatiotemporal framing accurately represents predation on large prey items (Beier et al. 1995; Knopff et al. 2009). Clusters were generated using the GPSeqClus package in R Studio (Clapp 2023; R Core Development Team 2024). We identified kill sites from GPS clusters using logistic regression models, which have been shown to successfully identify potential kill sites at clusters with > 90% field‐verified accuracy for large prey items (Clapp et al. 2022; Knopff et al. 2009, 2010; Smith 2014; Wilckens et al. 2016). To reduce the probability of false positives (i.e., putative kill sites where a deer was not actually killed), we only used clusters where GPS points were aggregated over a period > 45 h and there was a 95% + probability of being a kill site as estimated by following the modeling approach in Knopff et al. (2010) and J. B. Smith (2014). We removed one cluster that represented a den site (Boyce 2007). We checked other suspected denning clusters (> 10 days) by checking GPS data for a small home range centered around a den (Beier et al. 1995; Yovovich et al. 2020). To ensure no kill sites were double‐counted, we excluded locations that were within 1 km or 7 days of a putative cluster. GPS clusters were identified in areas where mule deer were the only large prey species, and depredation by pumas is relatively rare in San Diego County (< 134 reported between 1972 and 2019; Dellinger et al. 2021), meaning we were confident all identified GPS clusters represented mule deer kill sites. We also removed six GPS clusters identified in grazing land to further increase our confidence that all putative kill sites represented deer kill sites rather than livestock (California Department of Conservation 2025). Our final sample for dataset 3 included 97 putative deer kill sites.

From the full complement of datasets, we identified 211 bighorn kill sites, 142 deer kill sites, and 97 putative deer kill sites, totaling 450 kill sites across all three data sources. We then validated our technique as described in Appendix SA (Tables S1 and S2; Figures S1–S4).

### 2.5 Environmental Data

To understand how environmental factors may influence predation risk, we included five topographic covariates, four habitat covariates, one forage availability covariate, and season in our predation risk models (Cain et al. 2024; Dellinger et al. 2020; Lowrey et al. 2019; Peterson et al. 2021). To evaluate the impact of topography on predation risk, we used elevation (m), percent slope, aspect (degrees), topographic position index (TPI), and vector ruggedness measurement (VRM). Elevation was extracted from the U.S. Geological Survey (USGS) 30 m/1‐arc sec Digital Elevation Models (DEM; U.S. Geological Survey 2024). Slope, aspect, and TPI were calculated from USGS DEMs using QGIS (QGIS Development Team 2024), and VRM was calculated using R Studio and the *spatioEco* package (Evans and Murphy 2023; R Core Development Team 2024). To investigate the effect of vegetation, we included land cover type and percent canopy cover in our predation risk models. Land cover type included 13 major designations based on the California Wildlife Habitat Relationship classes (CALFIRE FRAP 2015). Canopy cover was sourced from the National Land Cover Database CONUS 2021 Tree Canopy Cover (USDA Forest Service 2023). To assess the impact of landscape features like roads and available water, we calculated the Euclidean distance to the nearest public road (Caltrans 2023) and perennial water source (Table 1). We combined a water bodies layer, a streams layer with flow probabilities, and locations of man‐made water sources and perennial springs to form a perennial water source layer, only including streams with an average monthly flow probability > 50% based on data from Zimmerman et al. (2018) (Table 1). We used two seasons to classify the timing of kill sites, a wet season from November 1st to April 30th, and a dry season from May 1st to October 31st. To capture the seasonality of food availability for both species, we calculated forage availability (based on NDVI) for each season (*n* = 2 seasons) per year for each year (*n* = 29 years) included in our kill site dataset. To create an NDVI raster for each combination of season and year (e.g., wet season 2000), we derived NDVI values from three Landsat images per season per year and averaged the resulting three NDVI rasters together. For more information, see Supplemental Document Appendix SB (Table S3).

**TABLE 1:** Environmental covariates, measures of prey density, data sources, and model elements.

Variable	Source	Bighorn model	Deer model	Combined model
Canopy cover (%)	National Land Cover Database CONUS 2021 Tree Canopy Cover (USDA Forest Service 2023)	x	x	x
Vegetation type (categorical)	California Department of Forestry and Fire Protection's Fire and Resource Assessment Program (CALFIRE FRAP 2015)	x	x	x
Distance to road (m)	CalTrans All Roads Linear Referencing Services (Caltrans 2023)	x	x	x
Distance to water source (m)	Water bodies layer (California Department of Fish and Wildlife 2013); Streams layer with flow probabilities (Zimmerman et al. 2018); Man‐made/perennial springs (Barrows and McGinnis 2014; California Department of Fish and Wildlife 2025)	x	x	x
Elevation (m)	USGS Digital Elevation Model (U.S. Geological Survey 2024)	x	x	x
Vector ruggedness measure (VRM)	USGS Digital Elevation Model (U.S. Geological Survey 2024)	x	x	x
Topographic Position Index (TPI)	USGS Digital Elevation Model (U.S. Geological Survey 2024)	x	x	x
Slope (%)	USGS Digital Elevation Model (U.S. Geological Survey 2024)	x	x	x
Aspect (Degrees)	USGS Digital Elevation Model (U.S. Geological Survey 2024)	x	x	x
Normalized Difference Vegetation Index (NDVI)	USGS Landsat 4, 5, 7, 8, and 9 Satellite Imagery (Earth Resources Observation and Science (EROS) Center 2025a, 2025b, 2025c)	x	x	x
Season (Wet: November 1st—April 30th or Dry: May 1st—October 31st)	(Tomaszewski et al. 2022)	x	x	x
Bighorn and Deer sympatric (Yes or No)	Public (County of San Diego 2016; GBIF.org 2025; San Diego Management and Monitoring Program 2016) Private (Jennings and Lewison 2013; Tremor et al. 2017; Center for Natural Lands Management, Unpublished Data) Government (Marine Corps Base Camp Pendleton, Unpublished Data; UC Davis & CDFW Puma GPS Data) Previous studies (Mitelberg and Vandergast 2016)			x
Deer habitat suitability	Public (County of San Diego 2016; GBIF.org 2025; San Diego Management and Monitoring Program 2016) Private (Jennings and Lewison 2013; Tremor et al. 2017; Center for Natural Lands Management, Unpublished Data) Government (Marine Corps Base Camp Pendleton, Unpublished Data) Previous studies (Mitelberg and Vandergast 2016)	x		
Bighorn habitat suitability	CDFW Peninsular Bighorn GPS Data, CDFW Helicopter Surveys, CDFW Ground Monitoring		x	

### 2.6 Prey Habitat Suitability and Spatial Extent

Because the robust data needed to construct density estimates for each of our prey species are lacking, we used habitat suitability as a proxy for relative abundance, as previous studies have demonstrated significant positive correlations between habitat suitability and abundance for a range of vertebrates, including birds, reptiles, amphibians, and mammals (Brambilla et al. 2024; De La Fuente et al. 2021; Weber et al. 2017). Prey habitat suitability was calculated using boosted regression trees built from a large dataset of field observations, supplemented by GPS collar data, and randomly sampled background points (Table 1). For each GPS‐collared individual, we randomly selected one location per year to prevent oversampling of collared individuals. Prey habitat suitability models were built using an equal number of background points as presence points (Barbet‐Massin et al. 2012), which were randomly generated across the full study area. For maps of deer and bighorn habitat suitability, see Appendix SB (Figures S5 and S6), and for the incorporation of an ecological neighborhood (Addicott et al. 1987) for prey habitat suitability, see Appendix SC (Tables S5 and S6; Figures S7–S10).

To estimate the spatial extent of deer and bighorn sympatry, we calculated a 95% kernel density estimator (KDE) from the deer observations that were used to estimate habitat suitability and overlaid it with the bighorn recovery region polygon and extracted the overlapped area to represent the region of deer and bighorn overlap. We also added a buffer to this overlap region to account for puma movement. Bighorn and deer that live close together without directly overlapping could experience a neighborhood effect if pumas can easily move between bighorn and deer‐only areas. Therefore, we added the buffer to represent the areas where a puma could access both deer and bighorn as prey sources. To calculate the buffer distance, we used the GPS locations from the 23 pumas in Dataset 3. We calculated core home range areas using 50% KDEs for each of the 23 pumas (Samuel et al. 1985). The average radius of the core areas for the 23 pumas was 6 km, which we added to the perimeter of the overlap region as the buffer.

### 2.7 Estimating Predation Risk

For the BRTs used to estimate predation risk, we used an equal number of absences and kill sites (Barbet‐Massin et al. 2012). We did not have true absence data that would traditionally be used for these analyses, so alternatively, we developed pseudoabsences where a kill was not observed. To do so, we buffered each kill site by 500 m, as seen in previous research (Cristescu et al. 2019; Ng'weno, Ford, et al. 2019), to ensure pseudoabsences weren't located near kill sites and randomly selected a GPS location from outside the buffers from data of extant GPS collared individuals. For more information on pseudoabsence point selection, see Appendix SB.

We validated each BRT using 5‐fold cross‐validation and evaluated predictive skill with area under the curve (AUC), true skill statistic (TSS), and mean absolute error (MAE; Allouche et al. 2006; Jiménez‐Valverde 2012). We tuned hyperparameters (number of trees, learning rate, minimum observations per node) by comparing AUC values for 54 different combinations of hyperparameters and selecting the model with the fewest number of trees within 0.005 AUC of the best model to balance model fit and computation time. For more information on tuning results and procedure, see Appendix SB (Table S4). We generated risk maps from each model across their respective spatial extent (Figure 1) using the *flexsdm* package (R Core Development Team 2024; Velazco et al. 2022). To account for the covariates that vary temporally (season, NDVI), we generated risk maps for each combination of season and year and then averaged them together to create a final risk map for each model. We assessed the importance of each covariate using relative importance values and how risk changed across values for each covariate using partial dependence plots (Almasieh et al. 2023).

## 3 Results

### 3.1 Bighorn Predation Risk Model

**FIGURE 3:**
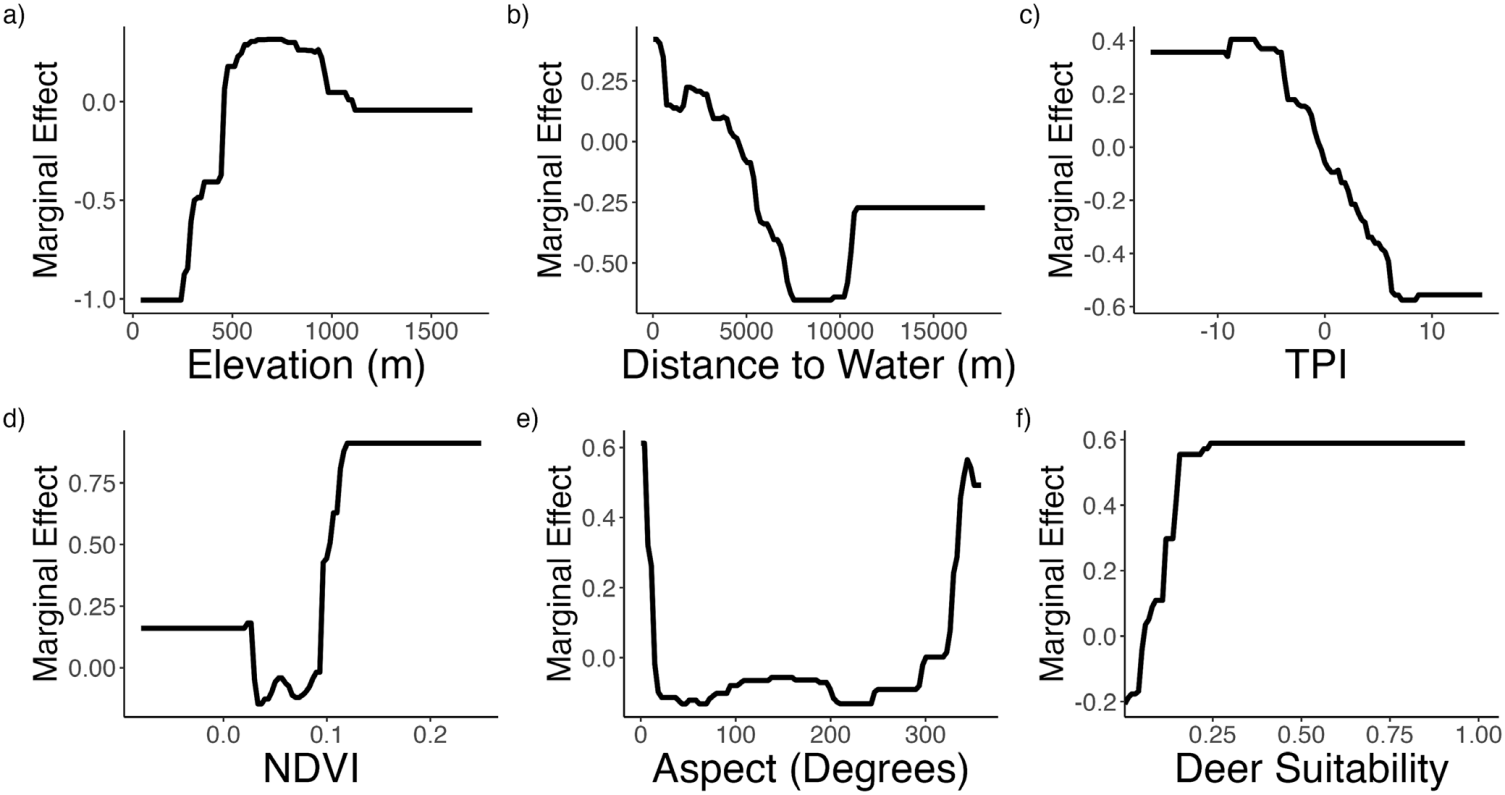
Partial dependence plots for the six most influential environmental covariates in the bighorn model. Environmental covariates are along the *x*‐axis, and the marginal effect on predation risk is along the *y*‐axis. Zero represents no effect on predation risk, positive values represent increases in predation risk, and negative values represent decreases in predation risk.

The bighorn predation risk model showed robust performance metrics (Table 2) and was most strongly influenced by elevation, followed by distance to water, TPI, NDVI, aspect, and deer habitat suitability (Table 3; Figure 6a). Model‐predicted predation risk rapidly increased from 0.27 to ~0.57 as elevation increased from 250 to 600 m, where risk was highest (Figure 3a; Figure S14). Model‐predicted predation risk also increased from 0.50 to 0.68 as deer habitat suitability increased marginally from 0 to 0.15 and risk also increased from 0.56 to 0.78 as NDVI increased from 0.08 to 0.12 (Figure 3d,f; Figure S14). In contrast, distance to water and TPI had inverse relationships with model‐predicted predation risk, with risk decreasing from ~0.66 to 0.44 as TPI increased from −4 to 6 and from ~0.65 to ~0.39 as distance to water increased from 0 to 7.5 km (Figure 3b,c; Figure S14). Predation risk was highest at 0.72 at north‐facing aspects compared to ~0.56 at all other aspects (Figure 3e; Figure S14, see Appendix SD for the remaining response curves, Figure S11).

**TABLE 2:** Performance metrics (AUC and TSS) for all three predation risk models.

	Bighorn predation risk model	Deer predation risk model	Combined predation risk model
AUC	0.733	0.805	0.755
TSS	0.414	0.514	0.416
MAE	0.429	0.347	0.397

**TABLE 3:** Relative influence values for the twelve covariates included in the three predation risk models.

Model	Prey suitability	Slope	Distance to water	NDVI	Distance to roads	TPI	Aspect	VRM	Elevation	Season	Land cover	Canopy
Bighorn	9.9	5.7	15.9	10.4	7.5	10.6	10.2	6.7	**16.9**	4.4	1.7	0.2
Deer	9.2	11.3	4.8	8.1	**15.7**	10.1	4.0	4.7	5.4	3.5	10.3	13.1
Combined	4.9	7.3	7.4	9.8	**15.9**	13.1	5.8	7.0	10.0	0.0	10.3	8.4

*Note:* Bold values indicate the most influential covariate for each model. Prey suitability is different for each model. For bighorn, it is deer suitability. For deer, it is bighorn habitat suitability. For “Combined”, it is binary bighorn and deer sympatry.

### 3.2 Deer Predation Risk Model

**FIGURE 4:**
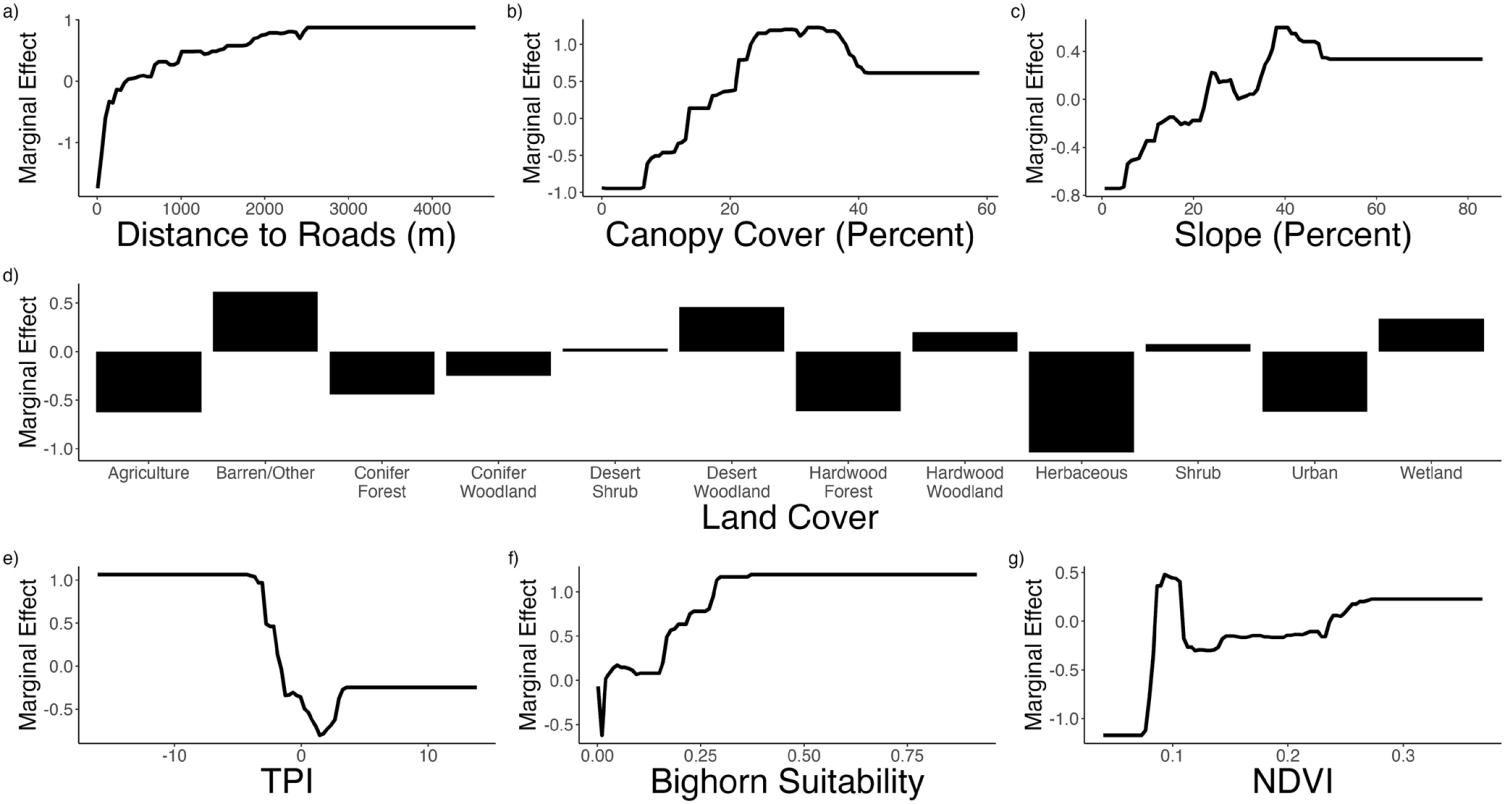
Partial dependence plots for the seven most influential environmental covariates in the deer model. Environmental covariates are along the *x*‐axis, and the marginal effect on predation risk is along the *y*‐axis. Zero represents no effect on predation risk, positive values represent increases in predation risk, and negative values represent decreases in predation risk.

The deer predation risk model showed robust performance metrics (Table 2) and was most strongly influenced by distance to roads, followed by canopy cover, slope, land cover, TPI, and bighorn habitat suitability (Table 3, Figure 6b). Model‐predicted deer predation risk increased with distance to roads, canopy cover, slope, and bighorn habitat suitability (Figure 4a–c,f). Specifically, model‐predicted risk increased from 0.4 to 0.9 as distance to roads increased from 0 to 2.5 km, from 0.65 to 0.94 as canopy cover increased from 0% to 25%, from 0.75 to 0.92 as slope increased from 0% to 38%, and from 0.81 to 0.94 as bighorn suitability increased from 0 to 0.30 (Figure S15). In contrast, TPI was the only covariate with an inverse relationship, with risk decreasing from 0.96 to 0.77 as TPI increased from −4 to 2 (Figure 4e; Figure S15). Barren cover (model‐predicted risk = 0.86), desert woodland (0.84), hardwood woodland (0.81), shrub (0.79), and desert scrub (0.78) were associated with higher model‐predicted predation risk, while hardwood forest (0.65), conifer forest (0.69), and herbaceous cover (0.55) were associated with lower model‐predicted predation risk (Figure 4d; Figure S15, see Appendix SD for the remaining response curves, Figure S12).

### 3.3 Combined Predation Risk Model

**FIGURE 5:**
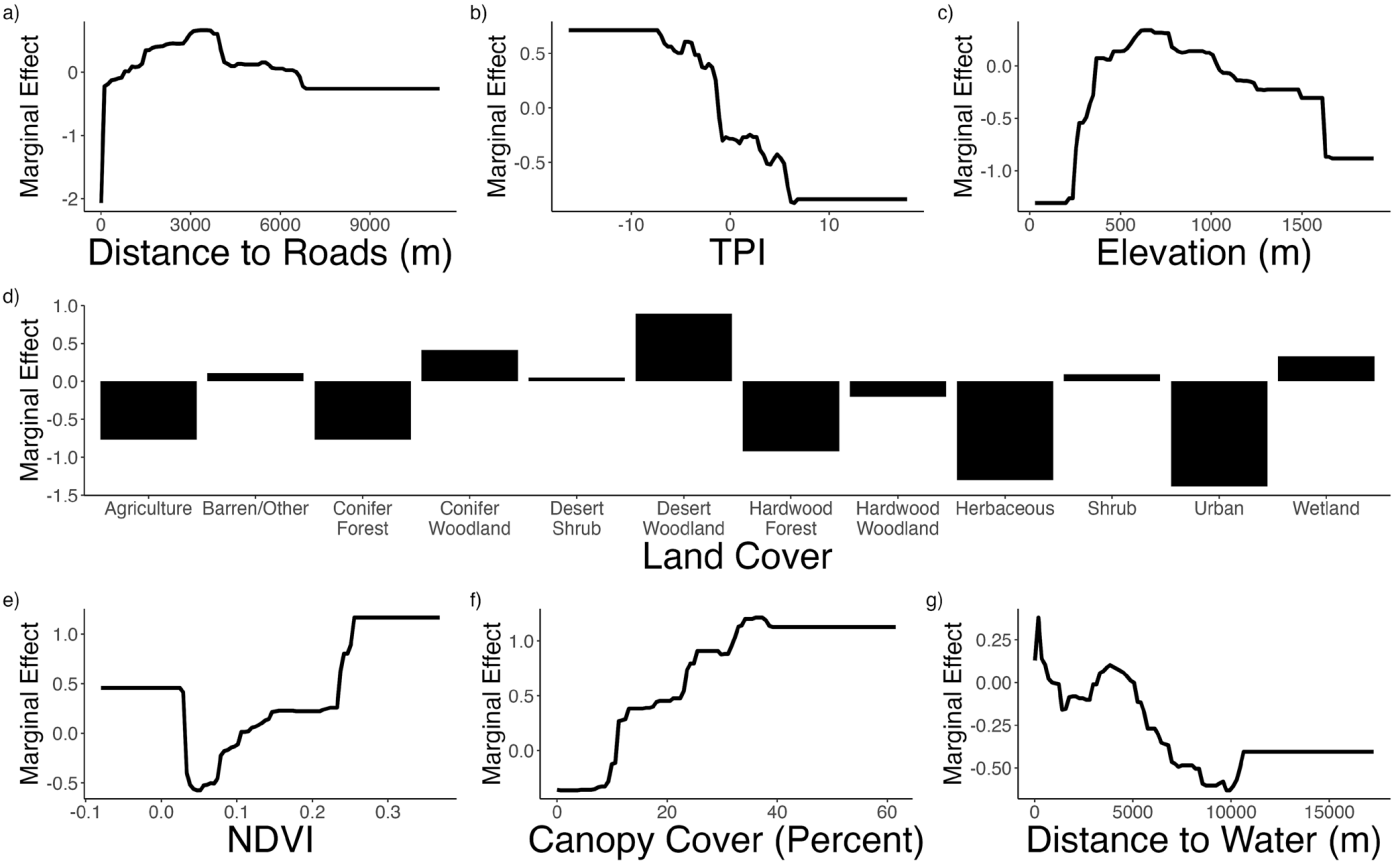
Partial dependence plots for the seven most influential environmental covariates in the combined predation risk model. Environmental covariates are along the *x*‐axis, and the marginal effect on predation risk is along the *y*‐axis. Zero represents no effect on predation risk; positive values represent increases in predation risk, and negative values represent decreases in predation risk.

**FIGURE 6:**
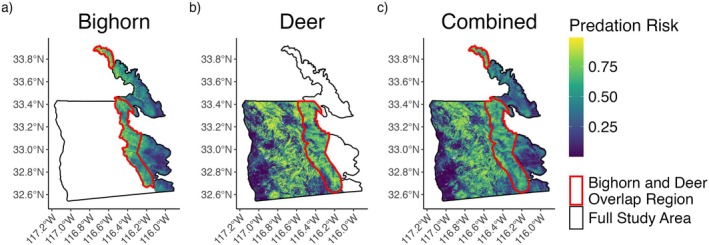
Maps of predation risk from pumas averaged across all years and both seasons for (a) bighorn, (b) deer, and (c) both species combined. Lighter colors (yellow and green) represent areas of higher predation risk, while darker areas (blue) represent areas of lower predation risk. The red outline marks the area where bighorn and deer spatially overlap, while the black outline is the full study area boundary.

The combined bighorn and deer predation risk model showed robust performance metrics (Table 2) and was most strongly influenced by distance to roads, followed by TPI, land cover, elevation, NDVI, and canopy cover (Table 3, Figure 6c). Model‐predicted predation risk was highest at intermediate values of distance to roads and elevation (Figure 5a,c). Specifically, model‐predicted predation risk was 0.82 at 3 km from roads compared to 0.65 at 100 m and 7 km from roads and 0.8 at an elevation of 800 m compared to 0.44 at elevations below 200 m and 0.54 at elevations above 1700 m. Model‐predicted predation risk for both species increased from 0.77 to 0.94 as canopy cover increased from 0% to 35%, increased from 0.65 to 0.91 as NDVI increased from 0.05 to 0.26, and decreased from 0.9 to 0.65 as TPI increased from −7 to 6 (Figure 5b,e,f; Figure S16). Barren cover (model‐predicted risk = 0.60), desert woodland (0.77), conifer woodland (0.67), desert scrub (0.59), and shrub (0.60) were associated with higher predation risk, while hardwood forest (0.35), conifer forest (0.39), and herbaceous cover (0.27) were associated with lower predation risk (Figure 5d; Figure S16, see Appendix SD for the remaining response curves, Figure S13).

## 4 Discussion

Spatial heterogeneity in predation risk is an influential factor in the conservation of managed and at‐risk prey species (Chitwood et al. 2022). In particular, sympatry between two prey species differing in abundance can elevate predation risk, presenting particular challenges for depleted species of conservation concern (DeCesare et al. 2010; Sinclair et al. 1998; Wittmer et al. 2005). In this case study of one apex predator and two sympatric ungulates with differing abundances, we found predation risk for each species was higher in areas of sympatry. For Peninsular bighorn sheep, an endangered species, any degree of sympatry with deer, even at lower levels of deer habitat suitability, increased predation risk (Figure 3f). Our findings suggest that deer abundance, as measured by habitat suitability, was associated with increased predation risk for bighorn, which could create challenges in the continued recovery of the species, particularly because pumas are also listed as species of conservation concern and southern mule deer are a subspecies of conservation interest.

### 4.1 Bighorn Predation Risk

Our results suggest that elevated predation risk for bighorn is driven by a neighborhood effect (Ng'weno, Ford, et al. 2019). In our study system, the neighborhood effect likely results from concentrated hunting by pumas in habitats shared by bighorn and deer, which could lead to opportunistic predation on bighorn. This effect would account for the higher predation risk for bighorn compared to areas where they are allopatric with deer.

As an endangered population, predation by pumas could have substantial impacts on the bighorn population (DeCesare et al. 2010; Johnson et al. 2013; Wittmer et al. 2005). Peninsular bighorn are non‐migratory and philopatric (Boyce et al. 1999; Rubin et al. 1998; U.S. Fish and Wildlife Service 2000), meaning different recovery regions likely experience varying rates of risk depending primarily on their proximity to deer. The disparity in risk for bighorn between the west side of their range where risk is higher and the east side where risk is lower (Figure 6a) may be important for future population models and to tailor management actions for each bighorn recovery region based on the level of risk in each region. Increased predation from pumas has been found to cause declines in lamb survival as well as declines in ewe mass gain in the summer and lamb mass at weaning (Cloutier et al. 2024). This suggests that bighorn coexisting with deer may experience reduced recruitment and poorer body condition, potentially contributing to population decline. Poor body condition leaves ewes susceptible to stressors (e.g., drought, predation, and disease), hindering their ability to raise lambs and resulting in decreased survival for both adults and their young (Bilodeau‐Hussey et al. 2025; Stephenson et al. 2020). Because ewes have been found to select neonatal lambing habitat with low predation risk (Forshee et al. 2022), ewes that are sympatric with deer may experience lower fecundity since there are fewer low‐risk areas. Given their endangered status, reduced recruitment due to increased predation could substantially limit the Peninsular bighorn population's ability to recover (Paterson et al. 2021).

Environmental factors, including topography, forage, and distance to water were the strongest drivers of predation risk. Lower elevations had the lowest risk for bighorn. Elevation within bighorn habitat generally decreases from west to east as the terrain shifts from the slopes of the Peninsular ranges to low elevation desert. Lower predation risk in the eastern portions of bighorn habitat, where elevation is lower, suggests that pumas may not select for habitat farther east, instead staying closer to the higher elevation forest and shrubland where the habitat may be more suitable and prey availability may be greater. Conversely, predation risk was higher in drainages and valley bottoms and lower at hilltops and ridgelines. Because visibility is lower in drainages and valley bottoms (Mistick et al. 2024) and pumas, as ambush predators, rely on cover to stalk prey, limited visibility in these areas contributes to increased kill success (Blake and Gese 2016; Karsch et al. 2016). Predation risk was also highest on north‐facing aspects. North‐facing slopes tend to receive more shade, increasing vegetation growth and cover, and providing more cover for pumas to ambush their prey (Cristescu et al. 2019). Resource distribution also influenced risk patterns. Proximity to water resulted in increased predation risk. This aligns with a previous study by Blake and Gese (2016) that found pumas feed on deer and bighorn closer to water sources, but is contrary to Jones et al. (2022), who found no impact of distance to water on risk when desert bighorn were examined individually. In dry, desert climates, surface water can be rare, causing species to congregate near limited water sources, which can lead to increased interactions between predators and prey (Glass et al. 2022; Harris et al. 2020). Finally, predation risk increased with increasing NDVI. Higher NDVI can be associated with lower visibility, increased puma presence, and higher predation risk (Ng'weno, Ford, et al. 2019; O'Malley et al. 2024; Smith et al. 2019). However, a previous study by Jones et al. (2022) found NDVI had no impact on desert bighorn predation risk. Peninsular bighorn live in desert environments where NDVI is relatively low, and these varying results suggest NDVI may not be a reliable proxy for visibility, and further research is needed to clarify its relationship with predation risk.

### 4.2 Deer Predation Risk

Our analyses also showed that spatial overlap in suitable habitat for bighorn and deer was associated with increased predation risk for deer but to a lesser extent. These results suggest that deer also experience a neighborhood effect, facing greater predation risk and mortality in sympatric habitats compared to allopatric ones. However, given that deer are more abundant than bighorn and they overlap with bighorn in a substantially smaller portion of their range, the magnitude and likely impact of the neighborhood effect are substantially lower for deer than for bighorn. This asymmetrical relationship between bighorn and deer is consistent with neighborhood effects and apparent competition in other predator and multi‐prey systems where the less abundant species is more negatively impacted compared to the more abundant species (DeCesare et al. 2010; Holt and Bonsall 2017; Johnson et al. 2013; Serrouya et al. 2015; Wittmer et al. 2005, 2007; Van Duyne et al. 2009). While deer are not endangered, they are a harvested species managed with sustainable hunting quotas, which may need to be adjusted for deer sympatric with bighorn to account for increased predation risk.

Deer predation risk was primarily influenced by topography, habitat, and distance to roads. Predation risk was lower in proximity to roads, which aligns with numerous studies that have shown pumas appear to select for areas farther from roads or human disturbance (Blake and Gese 2016; Dellinger et al. 2020; Nicholson et al. 2014; Peterson et al. 2021; Smereka et al. 2020). This pattern of lower predation risk closer to roads may represent an example of the human shield effect or prey refuge, when areas close to humans allow prey to avoid predators (Van Scoyoc et al. 2023). Steeper slopes increased predation risk for deer, suggesting that steeper slopes hinder the ability of deer to escape puma predation. However, trends in this relationship remain varied, with previous studies corroborating our results (Benson et al. 2016; Blake and Gese 2016), while others found the opposite effect (Cristescu et al. 2019), or no effect at all (Harvey et al. 2025). This suggests that for deer, the relationship between predation risk and slope may vary based on geography. Canopy cover and habitat type, two habitat variables related to visibility, played an especially important role in deer predation risk (Mistick et al. 2024). As with bighorn, predation risk for deer was highest in drainages or valley bottoms and lowest at plains, hilltops, or ridgelines. However, low‐visibility habitats, such as those with higher canopy cover and dense vegetation—desert woodland, conifer woodland, desert shrub, and shrub—were especially detrimental for deer. These habitats were associated with higher predation risk, a finding affirmed in previous studies (Benson et al. 2016; Blake and Gese 2016; Harvey et al. 2025; Laundré and Hernández 2003; Lehman et al. 2017). Interestingly, conifer forests and hardwood forests were associated with lower predation risk, even though they typically have higher canopy cover. This discrepancy could be due to a lack of understory vegetation in these forest habitats, which limits stalking cover for pumas.

### 4.3 Combined Predation Risk

Our combined predation risk model attempted to discern predation risk from pumas, regardless of the prey species. Based on our results, puma predation is generally associated with distances to roads, topography, and habitat. We saw similar patterns in the single species bighorn and deer models, with predation risk increasing as distance to roads and forage availability increased and within drainages and valley bottoms. Higher predation risk was also associated with shrubland and woodland areas while lower predation risk was associated with forest and herbaceous habitats, as with the individual bighorn and deer risk models. These results align with previous research on puma habitat as pumas generally select areas farther from human disturbance (Blake and Gese 2016; Dellinger et al. 2020; Nicholson et al. 2014; Peterson et al. 2021; Smereka et al. 2020) and are more successful at hunting in areas with greater cover, such as shrubland and woodland habitats and areas with greater forage availability and lower visibility, such as valley bottoms and drainages (Benson et al. 2016; Blake and Gese 2016; Harvey et al. 2025; Laundré and Hernández 2003; Lehman et al. 2017). This finding suggests that whereas the relative importance of these covariates may vary among individual prey species, the relationships and patterns are likely to persist.

### 4.4 Conclusions

Our findings are timely given the ongoing efforts to support bighorn population recovery, as the USFWS Peninsular bighorn sheep recovery plan lists predation as a key threat (U.S. Fish and Wildlife Service 2000). By identifying the impact of spatial overlap between deer and bighorn on predation risk, wildlife managers can better understand the threat predation posed to Peninsular bighorn by accounting for their proximity and sympatry with deer. Peninsular bighorn also face other concurrent threats, such as drought and disease, that can make them more susceptible to predation (U.S. Fish and Wildlife Service 2000). Disease and drought can reduce body condition (Besser et al. 2021; Brown et al. 2025), leading to decreased survival and increased vulnerability to predation (Stephenson et al. 2020). Therefore, bighorn exposed to both elevated predation risk and drought or disease could face a greater risk of mortality. These threats also vary spatially across the landscape, making it critical for future research to evaluate their cumulative impact to inform effective management (U.S. Fish and Wildlife Service 2000). Given the limited quantitative assessments of bighorn predation risk, results from this study can support a more comprehensive understanding of bighorn population dynamics and recovery. Pumas are, however, also a species of conservation concern, while southern mule deer are a subspecies of conservation interest, which means management decisions must consider the effects on all three species (Butler et al. 2015; Chasco et al. 2017). Predator or primary prey control is a common management approach for addressing apparent competition (Gibson 2006). However, in this species assemblage, successful management will need to balance risk reduction with sustaining healthy populations (Butler et al. 2015; Chasco et al. 2017). Our results demonstrate how sympatry and neighborhood effects influence predation risk, with significant impacts for depleted populations. Future studies in multi‐prey systems may need to account for and incorporate these effects when estimating the variability of predation risk across heterogeneous landscapes.

## Author Contributions

**Daniel Gordon:** conceptualization (equal), formal analysis (lead), investigation (equal), methodology (equal), visualization (lead), writing – original draft (lead), writing – review and editing (equal). **Rebecca L. Lewison:** conceptualization (equal), methodology (equal), writing – review and editing (equal). **Randy Botta:** conceptualization (equal), data curation (equal), investigation (equal), writing – review and editing (equal). **Winston Vickers:** data curation (equal), investigation (equal), writing – review and editing (equal). **Fernando Najera:** data curation (equal), investigation (equal), writing – review and editing (equal). **Megan Jennings:** conceptualization (lead), methodology (lead), writing – review and editing (equal).

## Funding

This work was supported by California Department of Fish and Wildlife (Q2250402).

## Conflicts of Interest

The authors declare no conflicts of interest.

## Supporting information


**Data S1:** Supporting Information.

**Data S2:** ece374160‐sup‐0002‐Supinfo2.docx.
**Appendix A**. Putative puma cluster validation methods and results.
**Table S1:** Comparison of the relative influence values for the twelve covariates included in the deer predation risk model between the model that includes putative kill sites and the model without putative kill sites.
**Table S2:** Comparison of the relative influence values for the twelve covariates included in the combined predation risk model between the model that includes putative kill sites and the model without putative kill sites.
**Figure S1:** Comparison of the partial dependence plots for the seven most influential environmental covariates in the deer model when putative kill sites are included (red line) and when putative kill sites (blue line) are removed. Environmental covariates are along the *x*‐axis, and the marginal effect on predation risk is along the *y*‐axis. Zero represents no effect on predation risk, positive values represent increases in predation risk, and negative values represent decreases in predation risk.
**Figure S2:** Comparison of the partial dependence plots for the additional environmental covariates that did not rank among the top covariates in the deer model when putative kill sites are included (red line) and when putative kill sites (blue line) are removed. Environmental covariates are along the *x*‐axis, and the marginal effect on predation risk is along the *y*‐axis. Zero represents no effect on predation risk, positive values represent increases in predation risk, and negative values represent decreases in predation risk.
**Figure S3:** Comparison of partial dependence plots for the seven most influential environmental covariates in the combined kill sites model when putative kill sites are included (red line) and when putative kill sites (blue line) are removed. Environmental covariates are along the *x*‐axis, and the marginal effect on predation risk is along the *y*‐axis. Zero represents no effect on predation risk, positive values represent increases in predation risk, and negative values represent decreases in predation risk.
**Figure S4:** Comparison of the partial dependence plots the additional environmental covariates that did not rank among the top covariates in the combined kill sites model when putative kill sites are included (red line) and when putative kill sites (blue line) are removed. Environmental covariates are along the *x*‐axis, and the marginal effect on predation risk is along the *y*‐axis. Zero represents no effect on predation risk, positive values represent increases in predation risk, and negative values represent decreases in predation risk.
**Appendix B**. Additional information regarding our environmental covariates and predation risk models.
**Table S4:** Shows the hyperparameters used for each of the three predation risk models after tuning and selection of the model with the fewest trees within 0.005 AUC of the top model.
**Figure S5:** Map of bighorn habitat suitability across the full study used as a covariate in our mule deer predation risk model. Lighter colors (yellow and green) represent areas of higher habitat suitability, while darker areas (blue) represent areas of lower habitat suitability.
**Figure S6:** Map of mule deer habitat suitability across the full study used as a covariate in our bighorn predation risk model. Lighter colors (yellow and green) represent areas of higher habitat suitability, while darker areas (blue) represent areas of lower habitat suitability.
**Appendix C**. Incorporation of an ecological neighborhood effect for prey habitat suitability.
**Table S5:** Comparison of the relative influence values for the twelve covariates included in the bighorn predation risk model between the model that includes an ecological neighborhood effect of deer habitat suitability by smoothing the deer habitat suitability raster, and when the ecological neighborhood is not considered.
**Table S6:** Comparison of the relative influence values for the twelve covariates included in the deer predation risk model between the model that includes an ecological neighborhood effect of bighorn habitat suitability by smoothing the bighorn habitat suitability raster, and when the ecological neighborhood is not considered.
**Figure S7:** Comparison of the partial dependence plots for the six most influential environmental covariates in the bighorn model when an ecological neighborhood effect of deer habitat suitability is included by smoothing the deer habitat suitability raster (blue line) and when the ecological neighborhood is not considered (red line). Environmental covariates are along the *x*‐axis, and the marginal effect on predation risk is along the *y*‐axis. Zero represents no effect on predation risk, positive values represent increases in predation risk, and negative values represent decreases in predation risk.
**Figure S8:** Comparison of the partial dependence plots for the additional environmental covariates in the bighorn model that did not rank among the top covariates when an ecological neighborhood effect of deer habitat suitability is included by smoothing the deer habitat suitability raster (blue line) and when the ecological neighborhood is not considered (red line). Environmental covariates are along the *x*‐axis, and the marginal effect on predation risk is along the *y*‐axis. Zero represents no effect on predation risk, positive values represent increases in predation risk, and negative values represent decreases in predation risk.
**Figure S9:** Comparison of the partial dependence plots for the seven most influential environmental covariates in the deer model when an ecological neighborhood effect of bighorn habitat suitability is included by smoothing the bighorn habitat suitability raster (blue line) and when the ecological neighborhood is not considered (red line). Environmental covariates are along the *x*‐axis, and the marginal effect on predation risk is along the *y*‐axis. Zero represents no effect on predation risk, positive values represent increases in predation risk, and negative values represent decreases in predation risk.
**Figure S10:** Comparison of the partial dependence plots for the additional environmental covariates in the deer model that did not rank among the top covariates when an ecological neighborhood effect of bighorn habitat suitability is included by smoothing the bighorn habitat suitability raster (blue line) and when the ecological neighborhood is not considered (red line). Environmental covariates are along the *x*‐axis, and the marginal effect on predation risk is along the *y*‐axis. Zero represents no effect on predation risk, positive values represent increases in predation risk, and negative values represent decreases in predation risk.
**Appendix D**. Partial dependence plots for the additional covariates included in our predation risk models and partial dependence plots transformed to show model‐predicted predation risk on the *y*‐axis.
**Figure S11:** Partial dependence plots for the additional environmental covariates in the bighorn model that did not rank among the top covariates. Environmental covariates are along the *x*‐axis, and the marginal effect on predation risk is along the *y*‐axis. Zero represents no effect on predation risk, positive values represent increases in predation risk, and negative values represent decreases in predation risk.
**Figure S12:** Partial dependence Plots for the additional environmental covariates in the deer model that did not rank among the top covariates. Environmental covariates are along the *x*‐axis, and the marginal effect on predation risk is along the *y*‐axis. Zero represents no effect on predation risk, positive values represent increases in predation risk, and negative values represent decreases in predation risk.
**Figure S13:** Partial dependence plots for the additional environmental covariates in the combined predation risk model that did not rank among the top covariates. Environmental covariates are along the *x*‐axis, and the marginal effect on predation risk is along the *y*‐axis. Zero represents no effect on predation risk, positive values represent increases in predation risk, and negative values represent decreases in predation risk.
**Figure S14:** Partial dependence plots for the environmental covariates in the bighorn predation risk model. Environmental covariates are along the *x*‐axis, and model‐predicted predation risk is along the *y*‐axis. These partial dependence plots are the same as the ones within the main text and those shown above, except that the *y*‐axis has been changed to show model‐predicted predation risk instead of marginal effect.
**Figure S15:** Partial dependence plots for the environmental covariates in the deer predation risk model. Environmental covariates are along the *x*‐axis, and model‐predicted predation risk is along the y‐axis. These partial dependence plots are the same as the ones within the main text and those shown above, except that the *y*‐axis has been changed to show model‐predicted predation risk instead of marginal effect.
**Figure S16:** Partial dependence plots for the environmental covariates in the combined predation risk model. Environmental covariates are along the *x*‐axis, and model‐predicted predation risk is along the *y*‐axis. These partial dependence plots are the same as the ones within the main text and those shown above, except that the *y*‐axis has been changed to show model‐predicted predation risk instead of marginal effect.

## Data Availability

Data and code for this manuscript are provided as Files S1.
