# Supplementary material for: Sympatry Increases Predation Risk for an Endangered Ungulate
Source: Ecol Evol. 2026 Aug 7;16(8):e74160. doi: 10.1002/ece3.74160 (PMC13451154; doi:10.1002/ece3.74160)
Supplement: Supplementary file 1 [file ECE3-16-e74160-s002.docx]

**Supplementary Material for “Sympatry Increases Predation Risk for an Endangered Ungulate”**

**Appendix A: Putative puma cluster validation methods and results**

We validated our putative kill sites by first comparing them with kill sites from Datasets 1 and 2, demonstrating that the criteria we outlined for selecting clusters can identify kill sites. Next, we predicted kill sites using GPS data from the pumas in Dataset 2 and compared those to kill sites from Datasets 1 and 2 to further demonstrate that our putative clusters can identify kill sites. These two comparisons identified 53 of our predicted kill sites that matched with known kill sites. Lastly, we ran our predation risk models without kill sites from Dataset 3 to determine if including those kill sites dramatically altered the patterns of predicted predation risk.

Supplementary Table 1: Comparison of the relative influence values for the twelve covariates included in the deer predation risk model between the model that includes putative kill sites and the model without putative kill sites.

|  | Slope | Distance to roads | Land cover | Canopy | TPI | Elevation | Bighorn Suitability | NDVI | Season | Distance to water | Aspect | VRM |
| --- | --- | --- | --- | --- | --- | --- | --- | --- | --- | --- | --- | --- |
| With Putative Kill Sites | 11.3 | **15.7** | 10.3 | 13.1 | 10.1 | 5.4 | 9.2 | 8.1 | 3.5 | 4.8 | 4.0 | 4.7 |
| Without Putative Kill Sites | 10.6 | **16.9** | 9.7 | 9.1 | 8.2 | 3.4 | 8.5 | 9.3 | 7.6 | 5.3 | 6.4 | 5.1 |
| Difference | -0.7 | 1.2 | -0.6 | -4.0 | -1.9 | -2.0 | -0.7 | 1.2 | 4.1 | 0.5 | -2.4 | 0.4 |

Supplementary Table 2: Comparison of the relative influence values for the twelve covariates included in the combined predation risk model between the model that includes putative kill sites and the model without putative kill sites.

|  | TPI | Land cover | Distance to roads | Canopy | Elevation | Bighorn and Deer Sympatry | Slope | NDVI | Distance to water | Aspect | VRM | Season |
| --- | --- | --- | --- | --- | --- | --- | --- | --- | --- | --- | --- | --- |
| With Putative Kill Sites | 13.1 | 10.3 | **15.9** | 8.4 | 10.0 | 4.9 | 7.3 | 9.8 | 7.4 | 5.8 | 7.0 | 0.0 |
| Without Putative Kill Sites | 13.9 | 7.8 | **14.3** | 5.2 | 9.8 | 6.2 | 7.6 | 7.5 | 10.1 | 9.3 | 7.5 | 0.7 |
| Difference | 0.8 | -2.5 | -1.6 | -3.2 | -0.4 | 1.3 | -0.3 | -2.3 | 2.7 | 3.5 | 0.5 | 0.7 |

Supplemental Figure 1: Comparison of the partial dependence plots for the seven most influential environmental covariates in the deer model when putative kill sites are included (red line) and when putative kill sites (blue line) are removed. Environmental covariates are along the x-axis, and the marginal effect on predation risk is along the y-axis. Zero represents no effect on predation risk, positive values represent increases in predation risk, and negative values represent decreases in predation risk.

Supplemental Figure 2: Comparison of the partial dependence plots for the additional environmental covariates that did not rank among the top covariates in the deer model when putative kill sites are included (red line) and when putative kill sites (blue line) are removed. Environmental covariates are along the x-axis, and the marginal effect on predation risk is along the y-axis. Zero represents no effect on predation risk, positive values represent increases in predation risk, and negative values represent decreases in predation risk.

Supplemental Figure 3: Comparison of partial dependence plots for the seven most influential environmental covariates in the combined kill sites model when putative kill sites are included (red line) and when putative kill sites (blue line) are removed. Environmental covariates are along the x-axis, and the marginal effect on predation risk is along the y-axis. Zero represents no effect on predation risk, positive values represent increases in predation risk, and negative values represent decreases in predation risk.

Supplemental Figure 4: Comparison of the partial dependence plots the additional environmental covariates that did not rank among the top covariates in the combined kill sites model when putative kill sites are included (red line) and when putative kill sites (blue line) are removed. Environmental covariates are along the x-axis, and the marginal effect on predation risk is along the y-axis. Zero represents no effect on predation risk, positive values represent increases in predation risk, and negative values represent decreases in predation risk.

**Appendix B: Additional information regarding our environmental covariates and predation risk models**

Supplemental Table 3: Thirteen different land cover types found in the CALFIRE FRAP land cover database.

| Vegetation Cover Type | Description |
| --- | --- |
| Agriculture | Less than 10% natural vegetation and area is under cultivation |
| Barren/Other | Less than 10% vegetative cover |
| Conifer Forest | Conifer Forest > 10% cover |
| Conifer Woodland | Conifer Woodland > 10% cover |
| Desert Shrub | Desert shrub vegetation > 3% and not conifer, hardwood, shrub |
| Desert Woodland | Desert Woodland vegetation > 3% and not conifer, hardwood, shrub |
| Hardwood Forest | Hardwood Forest > 10% cover and conifer < 25% |
| Hardwood Woodland | Hardwood Woodland > 10% and conifer < 10% |
| Herbaceous | Herbaceous >10% cover and tree/shrub < 10% cover |
| Shrub | Shrub > 10% cover and tree < 10% cover |
| Urban | Urban >10% cover and native tree/shrub/grass < 10% cover |
| Water | Water |
| Wetland | Wetland > 10% cover and not tree/shrub |

NDVI Calculation:

We calculated the normalized difference vegetation index (NDVI) in R Studio using bands 3 and 4 from Landsat 4, 5, and 7 satellite imagery and bands 4 and 5 from Landsat 8 and 9 satellite imagery (Earth Resources Observation and Science (EROS) Center, 2025a, 2025b, 2025c).

Pseudoabsence selection:

For mortalities of collared bighorn and deer, an equal number of pseudoabsence points was randomly selected from a random set of collared individuals, with only one pseudoabsence point selected from each individual bighorn or deer. For bighorn and deer mortalities found at investigated puma clusters and putative puma clusters, we calculated the 99% kernel density estimate (KDE) for the associated puma. We then randomly selected an equal number of pseudoabsence points to the number of known clusters. Pseudoabsence points for puma clusters were drawn from collared bighorn locations for the bighorn predation risk model and the compiled deer location data for the deer predation risk model.

Removal of kill sites from predation risk models:

One of the collared bighorn kills fell outside of the bighorn study area and was therefore removed from the bighorn predation risk model, while one of the putative deer kill sites fell within the bighorn study area and was removed from the deer predation risk model. Both kill sites were still included within the combined predation risk model.

BRT Tuning Grid and Resulting Model Parameters:

We used six values for the number of trees (1000, 2000, 3000, 4000, 5000, 10000), three values for the learning rate (0.001, 0.005, 0.01), and three values for the minimum number of observations per node (5, 10, 15) when tuning our models.

Supplemental Table 4: Shows the hyperparameters used for each of the three predation risk models after tuning and selection of the model with the fewest trees within 0.005 AUC of the top model

|  | Bighorn | Deer | Combined |
| --- | --- | --- | --- |
| Number of Trees | 1000 | 3000 | 3000 |
| Learning Rate | 0.1 | 0.1 | 0.1 |
| Min Obs per Node | 5 | 15 | 15 |

Bighorn and Mule Deer Habitat Suitability Maps:

Supplemental Figure 5: Map of bighorn habitat suitability across the full study used as a covariate in our mule deer predation risk model. Lighter colors (yellow and green) represent areas of higher habitat suitability, while darker areas (blue) represent areas of lower habitat suitability.

Supplemental Figure 6: Map of mule deer habitat suitability across the full study used as a covariate in our bighorn predation risk model. Lighter colors (yellow and green) represent areas of higher habitat suitability, while darker areas (blue) represent areas of lower habitat suitability.

**References:**

Earth Resources Observation and Science (EROS) Center. (2025a). *Landsat 4-5 Thematic Mapper Level-2, Collection 2* [Dataset]. U.S. Geological Survey. https://doi.org/10.5066/P9IAXOVV

Earth Resources Observation and Science (EROS) Center. (2025b). *Landsat 7 Enhanced Thematic Mapper Plus Level-2, Collection 2* [Dataset]. U.S. Geological Survey. https://doi.org/10.5066/P9C7I13B

Earth Resources Observation and Science (EROS) Center. (2025c). *Landsat 8-9 Operational Land Imager / Thermal Infrared Sensor Level-2, Collection 2* [Dataset]. U.S. Geological Survey. https://doi.org/10.5066/P9OGBGM6

**Appendix C: Incorporation of an ecological neighborhood effect for prey habitat suitability**

Range of effect methods:

To incorporate an ecological neighborhood effect for bighorn and deer habitat suitability into predation risk models, we smoothed bighorn and deer habitat suitability raster layers based on their home range sizes. When smoothing rasters, the value of each individual cell was calculated by averaging a defined number of neighboring cells. We used three different home range sizes (min, mean, and max) for each suitability layer to estimate the neighborhood effect. For deer, the three home range sizes were 0.5km^2^, 1.5km^2^, and 4.5km^2^ while for bighorn, the three home range sizes were 2.0km^2^, 41km^2^, and 130km^2^.

Supplementary Table 5: Comparison of the relative influence values for the twelve covariates included in the bighorn predation risk model between the model that includes an ecological neighborhood effect of deer habitat suitability by smoothing the deer habitat suitability raster, and when the ecological neighborhood is not considered

|  | Deer Suitability | Slope | Distance to water | NDVI | Distance to roads | TPI | Aspect | VRM | Elevation | Season | Land cover | Canopy |
| --- | --- | --- | --- | --- | --- | --- | --- | --- | --- | --- | --- | --- |
| Without Range of Effect | 9.9 | 5.7 | 15.9 | 10.4 | 7.5 | 10.6 | 10.2 | 6.7 | **16.9** | 4.4 | 1.7 | 0.2 |
| With Range of Effect | 10.4 | 8.4 | 14.5 | 11.6 | 7.2 | 10.8 | 8.9 | 5.9 | **16.3** | 4.3 | 1.6 | 0.1 |
| Difference | 0.5 | 2.7 | -1.4 | 1.2 | -0.3 | 0.2 | -1.3 | -0.8 | -0.6 | -0.1 | -0.1 | -0.1 |

Supplementary Table 6: Comparison of the relative influence values for the twelve covariates included in the deer predation risk model between the model that includes an ecological neighborhood effect of bighorn habitat suitability by smoothing the bighorn habitat suitability raster, and when the ecological neighborhood is not considered

|  | Slope | Distance to roads | Land cover | Canopy | TPI | Elevation | Bighorn Suitability | NDVI | Season | Distance to water | Aspect | VRM |
| --- | --- | --- | --- | --- | --- | --- | --- | --- | --- | --- | --- | --- |
| Without Range of Effect | 11.3 | **15.7** | 10.3 | 13.1 | 10.1 | 5.4 | 9.2 | 8.1 | 3.5 | 4.8 | 4.0 | 4.7 |
| With Range of Effect | 12.8 | **15.1** | 10.4 | 12.6 | 9.7 | 6.0 | 8.8 | 8.0 | 3.5 | 4.6 | 4.2 | 4.3 |
| Difference | 1.5 | -0.6 | 0.1 | -0.5 | -0.4 | 0.6 | -0.4 | -0.1 | 0.0 | -0.2 | 0.2 | -0.4 |

Supplemental Figure 7: Comparison of the partial dependence plots for the six most influential environmental covariates in the bighorn model when an ecological neighborhood effect of deer habitat suitability is included by smoothing the deer habitat suitability raster (blue line) and when the ecological neighborhood is not considered (red line). Environmental covariates are along the x-axis, and the marginal effect on predation risk is along the y-axis. Zero represents no effect on predation risk, positive values represent increases in predation risk, and negative values represent decreases in predation risk.

Supplemental Figure 8: Comparison of the partial dependence plots for the additional environmental covariates in the bighorn model that did not rank among the top covariates when an ecological neighborhood effect of deer habitat suitability is included by smoothing the deer habitat suitability raster (blue line) and when the ecological neighborhood is not considered (red line). Environmental covariates are along the x-axis, and the marginal effect on predation risk is along the y-axis. Zero represents no effect on predation risk, positive values represent increases in predation risk, and negative values represent decreases in predation risk.

Supplemental Figure 9: Comparison of the partial dependence plots for the seven most influential environmental covariates in the deer model when an ecological neighborhood effect of bighorn habitat suitability is included by smoothing the bighorn habitat suitability raster (blue line) and when the ecological neighborhood is not considered (red line). Environmental covariates are along the x-axis, and the marginal effect on predation risk is along the y-axis. Zero represents no effect on predation risk, positive values represent increases in predation risk, and negative values represent decreases in predation risk.

Supplemental Figure 10: Comparison of the partial dependence plots for the additional environmental covariates in the deer model that did not rank among the top covariates when an ecological neighborhood effect of bighorn habitat suitability is included by smoothing the bighorn habitat suitability raster (blue line) and when the ecological neighborhood is not considered (red line). Environmental covariates are along the x-axis, and the marginal effect on predation risk is along the y-axis. Zero represents no effect on predation risk, positive values represent increases in predation risk, and negative values represent decreases in predation risk.

**Appendix D: Partial dependence plots for the additional covariates included in our predation risk models and partial dependence plots transformed to show model-predicted predation risk on the y-axis**

Supplemental Figure 11: Partial dependence plots for the additional environmental covariates in the bighorn model that did not rank among the top covariates. Environmental covariates are along the x-axis, and the marginal effect on predation risk is along the y-axis. Zero represents no effect on predation risk, positive values represent increases in predation risk, and negative values represent decreases in predation risk.

Supplemental Figure 12: Partial dependence Plots for the additional environmental covariates in the deer model that did not rank among the top covariates. Environmental covariates are along the x-axis, and the marginal effect on predation risk is along the y-axis. Zero represents no effect on predation risk, positive values represent increases in predation risk, and negative values represent decreases in predation risk.

Supplemental Figure 13: Partial dependence plots for the additional environmental covariates in the combined predation risk model that did not rank among the top covariates. Environmental covariates are along the x-axis, and the marginal effect on predation risk is along the y-axis. Zero represents no effect on predation risk, positive values represent increases in predation risk, and negative values represent decreases in predation risk.

Supplemental Figure 14: Partial dependence plots for the environmental covariates in the bighorn predation risk model. Environmental covariates are along the x-axis, and model-predicted predation risk is along the y-axis. These partial dependence plots are the same as the ones within the main text and those shown above, except that the y-axis has been changed to show model-predicted predation risk instead of marginal effect.

Supplemental Figure 15: Partial dependence plots for the environmental covariates in the deer predation risk model. Environmental covariates are along the x-axis, and model-predicted predation risk is along the y-axis. These partial dependence plots are the same as the ones within the main text and those shown above, except that the y-axis has been changed to show model-predicted predation risk instead of marginal effect.

Supplemental Figure 16: Partial dependence plots for the environmental covariates in the combined predation risk model. Environmental covariates are along the x-axis, and model-predicted predation risk is along the y-axis. These partial dependence plots are the same as the ones within the main text and those shown above, except that the y-axis has been changed to show model-predicted predation risk instead of marginal effect.
